# A critical appraisal of mucormycosis in COVID- 19 patients in a tertiary care centre in India

**DOI:** 10.18502/CMM.2023.150667

**Published:** 2023-03

**Authors:** Sujata Raychaudhuri, Juhi Taneja, Jaseetha Sasidharan, Mukta Pujani, Kanika Singh, Tathagata Chatterjee, Manjula Jain, Rajkumar Chandoke, Anil Rai, Zafar Abbas, Asim Das

**Affiliations:** 1 Department of Pathology, ESIC Medical College and Hospital Faridabad, India; 2 Department of Microbiology, ESIC Medical College and Hospital, Faridabad, India; 3 Department of Ear, Nose, and Throat, ESIC Medical College and Hospital, Faridabad, India; 4 Department of Radio Diagnosis, ESIC Medical College and Hospital, Faridabad, India; 5 Department of Physiology, ESIC Medical college and Hospital, Faridabad, India

**Keywords:** Aspergillosis, COVID-19, India, Mucormycosis

## Abstract

**Background and Purpose:**

Morbidity and mortality of opportunistic fungal infections in COVID-19 patients are less studied and defined. The patients receiving immunosuppressive therapy, broad-spectrum antibiotics, corticosteroids, and invasive and non-invasive ventilation are the high-risk groups.

**Materials and Methods:**

The demographic profile as well as clinical and radiological findings of all the patients with COVID-19 suspected of Mucormycosis (MM) were recorded. The tissue samples from all the patients were sent for microbiological (KOH mount and culture) and histopathological analysis for confirmation of MM.

**Results:**

In total, 45 COVID-19 patients suspected of MM were included in the study and MM was confirmed in 42 patients. The mean age of the patients was 50.30±14.17 years with a female: male ratio of 1.1:1. The most common symptom was headache (52.38%) followed by purulent nasal discharge (38.09%) and facial pain in 33.33% of the cases. The ocular symptoms included a diminution of vision (33.33%) and redness of the eye (2.38%). The most common site of involvement was rhino-orbital (42.85%) followed by sinonasal (23.80%) and rhino cerebral (19.04%). Majority (38.09%) of the patients were diagnosed with stage II of Rhino-orbital-cerebral Mucormycosis (ROCM) based on radiology. A history of diabetes mellitus and steroids was present in 97.61% and 85.71% of the cases, respectively. Moreover, KOH was positive for MM in 97.61% of the cases while the culture was positive in only 35.71% of the cases. In addition, on histopathology, MM was confirmed in 64.28 % of the cases. Mixed growth with *Aspergillus* species and *Rhizopus* species was observed in 14.28% of the cases in culture and 11.90% of the cases in histopathology test. Furthermore, angioinvasion was found in 23.80% of the cases according to the histopathology test.

**Conclusion:**

Based on the results, the most common conditions associated with MM in COVID-19 patients were diabetes mellitus and steroid therapy. A high level of clinical suspicion aided with diagnostic tests, including KOH mount, culture, histopathology, and radiology which helped the early detection of opportunistic fungal infection in COVID-19 patients to ensure timely treatment.

## Introduction

Coronavirus 2 (SARS-CoV-2) is implicated as the pathogenic virus causing Coronavirus disease 2019 (COVID-19) [ 1 ]. A wide range of opportunistic fungal infections, especially those caused by *Candida*, *Aspergillus*, and *Mucormycetes*, have been reported to be associated with COVID-19 [ 2 ]. Mucormycosis (MM) which is caused by the fungus of the order *Mucorales*, class *Mucormycetes*, and various genera, like *Rhizopus*, *Mucor*, *Rhizomucor*, *Cunninghamella*, and *Lichtheimia*. The MM is a rare angioinvasive and fatal disease affecting patients with diabetic ketoacidosis [ 3 ].

Prevalence of MM in India has been reported at 0.14 cases per 1000 population [ 4 ]. Moreover, there is an increase in the incidence rate of infections caused by the fungi of the order *Mucorales* in COVID-19 patients across the globe and especially in India. In this regard, the Indian government was prompted to declare it an epidemic and a notifiable disease. Recent data suggest that the prevalence of mucormycosis is 70 times higher in India, compared to the developed countries [ 5 ]. 

*Rhizopus arrhizus* is responsible for most of the cases of MM [ 5 ] and rhino-orbital-cerebral mucormycosis (ROCM) was the most common presentation in 90% of the cases [ 6 , 7 ]. The presentations of MM can include pulmonary, gastrointestinal, cutaneous, renal, and disseminated infections [ 6 , 7 ]. The major risk factors of MM in India have been reported to be diabetes mellitus (DM) and corticosteroid therapy [ 8 , 9 ]. In COVID-19 patients, various factors, such as hypoxia, pre-existing diabetes mellitus, steroid-induced new-onset diabetes, metabolic acidosis, and diabetic ketoacidosis (DKA), are all implicated as risk factors of MM [ 10 ].

Increased levels of ferritin and desferrioxamine (therapy for iron overload) are known to help fungi thrive by capturing the iron by siderophages [ 10 , 5 ]. The unique feature of MM is its damage to the vessel wall caused by extensive angioinvasion resulting in vascular thrombosis and necrosis of the surrounding tissues [ 11 ]. The COVID-19 infection which is caused by a Ribonucleic acid (RNA) virus is known to cause severe lymphopenia in the early stages, while in the later stages with an increased rate of viral replication, the epithelial endothelial barrier is deranged, leading to the influx of monocytes and neutrophils [ 12 ]. Dysfunction of endothelial cell barrier along with disruption of alveolar-capillary transport of oxygen results in reduced diffusing capacity of oxygen, which is a hallmark of COVID-19 infection [ 13 ]. 

This study aimed to examine the demographical, clinical, radiological, pathological, and microbiological findings of MM patients and analyze the risk factors associated with it.

## Materials and Methods

This study was conducted in a tertiary care hospital in Northern India in the Departments of Pathology and Microbiology over 3 months. This research included all suspected cases of MM after or during their COVID-19 infection (confirmed by reverse transcription polymerase chain reaction [RT-PCR]) as well as those with the presentation of one or more of the symptoms, like decreased/loss of vision, sinusitis, headache, facial cellulitis, diplopia, proptosis, toothache, loosening of teeth, blackish discoloration over the bridge of nose/palate, prolonged fever, jaw involvement, altered mental status, and necrosis of tissue with black crusts who were admitted to the hospital [ 14 , 15 , 16 ].

The demographic data and clinical details of the patients were recorded. Moreover, the clinically suspected MM patients were subjected to imaging, including computerized tomography scans (chest, head, and neck) and magnetic resonance imaging. The patients with ROCM were classified into four categories based on radiological classification by Honavar et al. [ 15 ]. The tissue biopsy samples were sent for histopathology, potassium hydroxide (KOH) (Sigma Aldrich, USA) and culture and Lacto phenol Cotton blue mount (LPCB, Himedia Mumbai, India). The biopsy specimens were processed and sections were stained with routine hematoxylin and eosin (H&E, Merck, USA) stain along with periodic acid-Schiff (PAS, Merck, USA) for fungus. 

Only the confirmed cases of MM either on histopathological or microbiological examination during the outbreak of COVID-19 were included in the study, while those whose fungal infection was not confirmed on microbiological or histo-pathological tests were excluded [ 17 ].

### Ethical Clearance

Ethical clearance was taken from the institutional ethical committee. (134X/11/13/2021-IEC/6 dated 4 June 2021).

### Statistical analysis

All the data, including the demographic characteristics and the clinical details of the patients, were tabulated. The continuous data were presented as mean and standard deviation. Moreover, the results were expressed in percentages. It should be noted that a p-value of < 0.05 was considered statistically significant.

## Results

In total, this study included 45 patients who were admitted to our institution with suspected MM. COVID pneumonia was found in 35.71% (n=15) of the cases with positive RT-PCR SARS CoV-2 test. The MM was confirmed in 42 patients either on microbiology or histopathology; consequently, the three cases that were negative for MM were excluded from the study. Both genders were almost equally affected with slight female preponderance with a female-to-male ratio of 1.1:1. The mean age of the patients was 50.3014.17 years (Table 1). Moreover, the majority of patients (28.57%) were within the age range of 51-60 years. 

**Table 1:** Demographic characteristics of patients

Gender	
Male	23 (54.76%)
Female	19 (45.23%)
**Age (years)**
20-30	4 (9.52%)
31-40	8 (19.04%)
41-50	8 (19.04%)
51-60	12 (28.57%)
61-70	7 (16.66%)
71-80	2 (4.76%)
81-90	1 (2.38%)

The patients with DM were found to be highly susceptible to COVID-associated MM with a positive percentage of 97.61% (*P=0.0001*) (Table 2). It is noteworthy that one patient also had DKA and another patient was on chemotherapy for acute lymphocytic leukemia. Besides, one patient was suffering from tuberculosis and was on anti-tubercular treatment. Furthermore, steroids were administered to 85.71% (n=36) of the patients which were also found to be significantly associated with MM (*P<0.0001*) (Table 2). In addition, a significant association was found between MM and oxygen therapy in COVID patients (*P=0.0024*) as 66.66% of cases (n=28) received oxygen therapy (Table 2). The mean duration of COVID-19 diagnosis and MM detection in patients was 33.2111.71 days. Moreover, hypertension and cardiovascular disease were observed in 10 (23.8%) and 13 (30.95%) out of 42 patients respectively. 

**Table 2:** Association of predisposing factors with COVID-associated mucormycosis

Factors	Number (percentage)	*P value*
Diabetes mellitus	41/42 (97.61%)	0.0001
Steroid intake	36/42(85.61%)	<0.0001
Oxygen therapy	28/42 (66.66%)	0.0024

The most common complaint was a headache, followed by purulent nasal discharge and facial pain as reported in 52.38%, 38.09%, and 33.33% of the patients, respectively (Table 3).

**Table 3:** Clinical symptoms in patients of mucormycosis

Symptom	Number of Cases	Percentage
Headache	22	52.38%
Purulent discharge from nose	16	38.09%
Facial pain	14	33.33%
Diminution of vision	14	33.33%
Nasal blockage	6	14.28%
Facial swelling	3	7.14%
Facial numbness	3	7.14%
Redness of eye	1	2.38%
Discharge from the middle ear	1	2.38%

In addition, a nasal blockage was reported in 11.90% of the patients while facial swelling and numbness over the face were noted in 7.14% of them. The ocular symptoms included a diminution of vision (33.33%) and redness of the eye (2.38%). It should also be mentioned that a single case reported discharge from the middle ear (2.38%) (Table 3). Most of the patients reported more than one symptom, of which headache and purulent nasal discharge were the most common (28.57%, n=12). 

**Figure 1:**
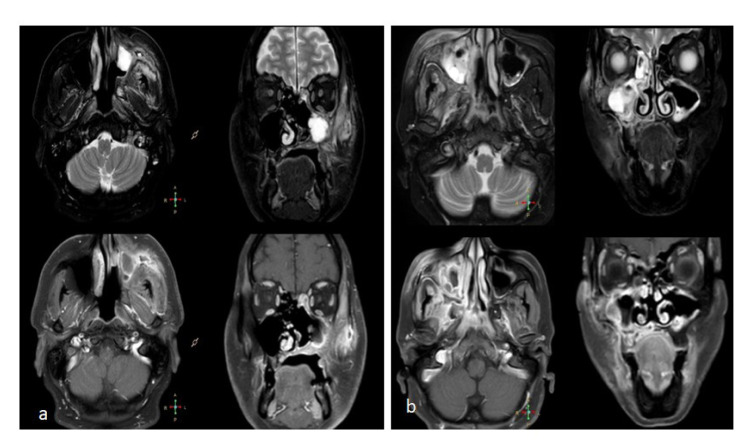
A) Axial and coronal T2 fat Sat and T1 contrast-enhanced magnetic resonance imaging (MRI) images showing enhancing polypoidal left maxillary sinusitis with pre-antral, retro-antral, and left facial soft tissue inflammatory changes. There is an extension along the left nasolacrimal duct. B) Axial and coronal T2 fat Sat and T1 contrast-enhanced MRI images showing enhancing polypoidal bilateral maxillary (right and left) and right ethmoid sinusitis with right pre-antral, retro-antral, and facial soft tissue inflammation. There is an intra-orbital extension along the floor of the right orbit.

Based on the radiological classification by Honavar et al. (Table 4), the majority of the patients with ROCM were classified as stage II (38.09%) while stage III and IV comprised 11 patients each (26.09%). The most common site of involvement by MM was the sinonasal area (Figure 1a) which was observed in 47.61% (n=20) of the patients followed by rhino-orbital involvement in nine patients (21.42%) (Figure 1b).

**Table 4:** Radiological classification of rhino-orbital-cerebral mucormycosis

Staging of rhino-orbital-cerebral mucormycosis	Symptoms	Signs
**Stage 1:** Involvement of the nasal mucosa	Nasal stuffiness/discharge, foul smell, and epistaxis	Black-tinged mucoid, hemorrhagic nasal discharge, and eschar
**1a:** Limited to the middle turbinate		
**1b:** Involvement of the inferior turbinate or ostium of the nasolacrimal duct		
**1c:** Involvement of nasal septum		
**1d:** Bilateral nasal mucosal involvement		
**Stage 2:** Involvement of paranasal sinuses	Symptoms of stage 1+facial pain, facial edema, dental pain, and systemic symptoms (i.e., fever and malaise)	Signs of stage 1+unilateral or bilateral, localized or diffuse facial edema, edema localized over the sinuses, and localized sinus tenderness
**2a:** One sinus		
**2b:** Two ipsilateral sinuses		
**2c:** More than two ipsilateral sinuses and/or palate/oral cavity		
**2d:** Bilateral paranasal sinus or involvement of the zygoma or mandible		
**Stage 3:** Bilateral orbital involvement	Symptoms in stages 1 and 2+pain in the eye, proptosis, ptosis, diplopia, and loss of vision	Signs in stage 1 and 2+conjuctival chemosis, restricted ocular motility, paraesthesia, ptosis, and proptosis
**Stage 4:** Involvement of the central nervous system	Symptoms of stages 1-3+bilateral proptosis, paralysis, and altered consciousness	Signs of stages 1-3+ hemiparesis
**4a:** Focal or partial cavernous sinus or cribriform plate		
**4b:** Diffuse cavernous sinus involvement		
**4c:** Involvement beyond cavernous sinus, skull base, and brain infection		
**4d:** Multifocal central nervous system disease		

The content is adapted from Honavar [ 15 ].

It must be mentioned that only 16.66% (n=7) of the patients showed rhino-cerebral involvement. Moreover, rhino-orbital-cerebral involvement was observed in 9.52% (n=4) of the cases while orbital involvement alone was observed in only 4.76% (n=2) of the cases. The maxillary sinus was the most common sinus involved with MM. Bony erosion involving the palate, maxillary sinus, clivus, pterygoid, or orbital bone by MM was noted in 23.80% of the cases (n=10). Intracranial involvement in the form of intracranial thrombosis was also seen in 0.09% (n=4) of the cases.

**Figure 2:**
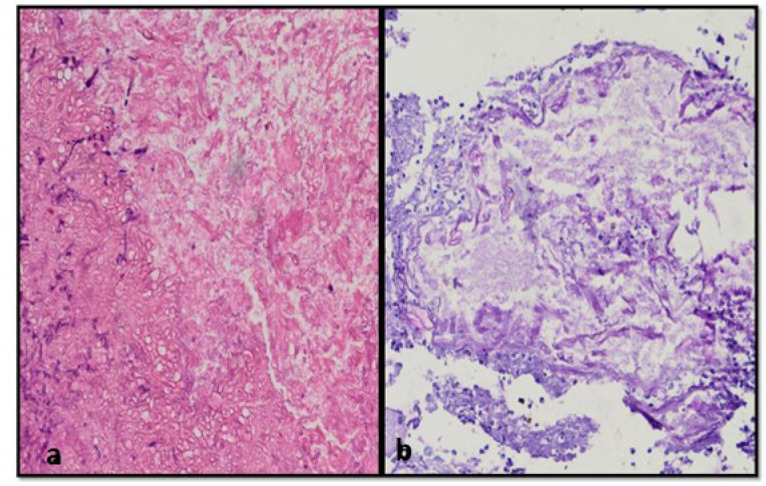
A) Hematoxylin and Eosin (H&E) stained sections show numerous broad aseptate fungal hyphae of Mucormycosis (100x). B) Periodic acid-Schiff-stained sections show fungal hypha in dark pink color.

**Figure 3:**
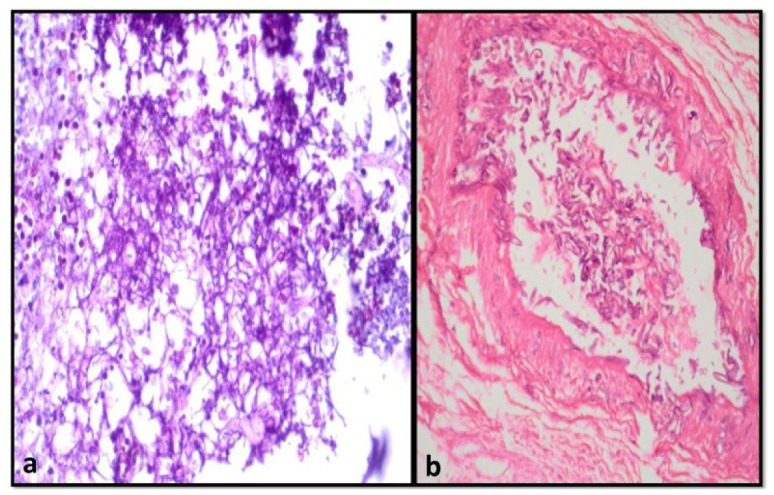
A) Hematoxylin and eosin (H&E) stained sections show a mixed infection of septate acute angle branching hyphae of Aspergillus along with broad aseptate hyphae of Rhizopus. B) The H&E-stained sections show the invasion of the vascular wall and lumen filled with broad aseptate fungal hyphae of mucormycosis (100x).

The KOH was highly sensitive as 41 out of 42 cases (97.61%) were positive for MM; however, the culture was positive only in 15 cases (35.71%). The cultures showing mixed growth of aspergillosis and MM were observed in six cases (14.28 %). Final identification of the fungi was carried out on LPCB made from the slide culture method. The histopathological examination was positive for MM in 27 cases (64.28%) (Figure 2). The PAS stain was used to confirm the presence of fungal infection in all the cases that were positive in the histopathology test (Figure 2). Five cases (11.90%) showed the presence of septate hyphae with dichotomous branching in addition to broad aseptate hyphae in histopathology (Figure 2). Only one case which was negative both in KOH and culture was found to be positive in histopathology. Angio-invasion was seen in 10 cases in histopathology (23.80%) (Figure 3, Table 1).

Intravenous liposomal amphotericin B was started in all patients after laboratory confirmation. Endonasal debridement was performed in all patients after confirmation of MM. Orbital exenteration was performed on four patients while maxillectomy along with endonasal debridement was performed on four patients as well. It should be mentioned that 3 out of the 42 patients expired due to complications arising out of MM.

## Discussion

There was no significant gender-based difference between patients in the present study; however, there was a slight female preponderance as the female-to-male ratio was 1.1:1. Selarka et al. [ 18 ] and Singh et al. [ 10 ] in their studies showed male predominance with prevalence rates of 74.5% and 78.9%, respectively. In the studies performed by Selarka et al. and Ravani et al., the mean age of participants was 55±12.8 and 56.3 years, respectively [ 18 , 19 ]. In the present study, the mean age of patients was 50.30±14.17 years.

The MM has a strong association with poorly controlled diabetes. Based on the results of a study conducted by Cornely et al., India ranks as the second country worldwide regarding the prevalence of adult diabetes in the age group of 20-79 years [ 17 ]. Results of the present study showed the association of MM with diabetes and DKA in 97.6% and 2.38% (n=1) of the participants, respectively. Findings of a study performed by Singh et al. revealed DM in 80% of cases and DKA in 14.9% of cases [ 10 ]. In another study carried out by Selarka et al., the incidence rate of DM was 76.6% [ 18 ]. Chavan et al. also reported DM as the most common risk factor with a percentage of 86.6% [ 20 ]. 

The ROCM is typically known to develop in patients with uncontrolled diabetes [ 18 ]. Diabetic patients have been reported to receive corticosteroids for the management of COVID pneumonia which leads to a state of hyperglycemia. Uncontrolled diabetes and steroid use lead to similar outbreaks as reported in other parts of the World [ 16 ].

According to Singh et al., individuals with hematological and other malignancies, patients with solid organ transplantation, and those on immunosuppressive or corticosteroid therapy are also at a higher risk of infection by *Mucor circinelloides* [ 10 ]. Based on a study performed by Peterson et al., endothelial cell dysfunction and inflammation with vasoconstriction in various organs put diabetic patients at increased risk of endotheliitis and accentuate the cytokine storm. Increased vasoconstriction can lead to organ ischemia, tissue oedema, and eventually, a procoagulant state [ 21 ]. 

A long course of steroid therapy has been implicated as a predisposing factor for opportunistic fungal infection. It should be mentioned that a long course of therapy consists of a progressive dose greater than 600 mg of prednisolone or a total of 2-7 mg of methyl prednisone administered for a month. A short course of steroid therapy in those with DM is also known as a predisposing factor for MM [ 10 ]. In the present study, 85.7% of the patients who developed MM were administered steroids. In a study performed by Singh et al., glucocorticoids were given to 76.3% of the cases who developed MM [ 10 ]. Ravani et al. reported DM in 96.7% of the cases and steroid use in 61.2% of the cases [ 19 ].

In a study carried out by Selarka et al., the factors contributing to the pathogenesis and virulence of MM included rapid growth, the ability to utilize host iron for growth, adherence to the endothelial surface, and downregulation of genes responsible for host defense [ 18 ]. Singh et al. found that the incidence rate of MM is high in patients with iron overload, hemochromatosis, des-ferrioxamine therapy, severe burns, AIDS, intravenous drug abuse, malnutrition, and open wounds following trauma [ 10 ]. 

Alteration of iron metabolism in addition to hyper-glycemia is present in severe COVID-19 cases; therefore, higher ferritin levels are reported among them. Due to higher ferritin levels, reactive oxygen species are generated which causes tissue damage and the release of free iron into circulation which acts as a risk factor for the development of MM [ 10 ]. The present study included one case with tuberculosis while all of the cases were on immunosuppressive therapy and developed MM.

All around the globe, ROCM is the most common clinically observed variety of MM [ 10 ]. The ROCM includes diseases ranging from limited sino-nasal involvement (sino-nasal tissue invasion) to limited rhino-orbital disease (progression to orbits) and rhino-orbital-cerebral disease (central nervous system involvement) [ 21 ]. In the present study, it was found that the major site involved was sinonasal (47.61%) followed by rhino-orbital (21.42%) and rhino-cerebral involvement (16.66%). The ROCM was observed in 9.52% of the cases, whereas orbital involvement alone was seen in only 4.76% of the cases. However, in research performed by Singh et al., the most common organs involved with MM were the nose and sinus (88.9%) followed by rhino-orbital (56.7%) and ROCM type (22.2%) [ 10 ]. John et al. in their study reported maximum cases of rhino-orbital (41%) followed by rhino-orbital-cerebral (27%) [ 22 ]. 

Radiologically, the majority of the patients in the present research were classified at stage II (38.09%) according to the staging proposed by Honavar et al. [ 15 ]. The most common symptom noted in the present study was headache (52.38%) followed by a purulent discharge from the nose (38.9%) and facial pain (33.33%) while ocular symptoms, like redness of eyes and diminution of vision, were noted in 33.33% of the cases. 

Results of this research are in line with those of the study performed by Selarka et al. [ 18 ] which indicated nonspecific headache (74.5%) as the major symptom. Other symptoms included disturbances in vision, diplopia, numbness, and facial weakness while a fraction of cases also had features of the weakness of eye muscles, damage to the optic nerve, and protrusion of the eye [ 18 ]. One patient also complained of ear discharge suggesting the involvement of the middle ear.

Singh et al. in their study indicated that verification of the fungus is performed by the identification of the hyphae based on width, septations, acute or obtuse angle branching, and pigmentations [ 10 ]. In the present study, septate hyphae could be demonstrated with dichotomous branching along with a few broad non-septate hyphae in the tissue. The fungal hyphae invade the vasculature causing fungal thrombosis, ischemia-induced infarction, and eventually, necrosis of the affected tissues [ 18 ]. 

Vaidya et al. showed that co-infection due to aspergillosis and MM in sinuses is a rare entity and difficult to diagnose [ 23 ]. Singh et al. have reported 10 cases of mixed infection with MM and aspergillosis, where all the patients were diabetic and seven patients had a history of steroid intake. In the aforementioned study, all 10 patients presented with similar complaints, such as headache, nasal obstruction, and discharge as well as eye symptoms, like pain and orbital swelling. In the present study, similar features were found in the six cases of mixed growth consisting of *R. arrhizus* and *Aspergillus* spp. was reported in six cases in culture and five cases in histopathology [ 14 ]. 

The MM damages the vascular wall leading to angioinvasion and thereby, vascular thrombosis and necrosis of surrounding tissue which was also noted in a study conducted by Mishra et al. [ 12 ]. It should be mentioned that angioinvasion was found in 23.80% of the cases in histopathology in the present research. 

According to the screening of multiple samples from each patient following debridement, the sensitivity of the KOH mount was high. Mohanty et al. in their study have reported the maximum (12/186) and minimum cases (01/186) of MM to be positive on KOH mount alone and biopsy samples, respectively [ 24 ]. It should be noted that sensitivity may vary between different tests as different sections of the sample are examined by each laboratory. In other words, the sample sent to the microbiology and histopathology departments may not be from the exact same site and thus not equally representative. Besides, sometimes it is very difficult to diagnose fungal infections on scanty tissue sections [ 25 ]. 

In the present study, confirmation of MM was performed by histopathological demonstration of tissue invasion in tissue sections obtained endoscopically and stained with H&E and PAS. Biopsy remains the gold standard for the diagnosis of MM [ 26 ]; however, the cases that were negative on histopathology but positive on microbiological examination were treated similarly to MM when there was a strong clinical and radiological suspicion since it is a life-threatening condition. Histopathology helps to confirm whether the fungal colonies in direct microscopy or culture are pathogenic or contaminant as well as invasive or noninvasive in nature. Angioinvasion was reported in 23.80% of the cases in this study [ 24 ].

In the present study, mortality was seen in three patients (7.14%). Another study has also reported mortality in 9.6% of the patients [ 19 ]. At our institution, orbital exenteration was performed in four patients to control the spread of infection.

## Conclusion

In developing countries, such as India, the most common conditions associated with MM are diabetes and steroid therapy. Opportunistic fungal infection in COVID-19 patients require early detection by imaging studies, direct microscopic examination, histopathology, and early surgical intervention to modify the aggressive course of the disease. Strict glycemic control and cautious use of glucocorticoids in patients with COVID pneumonia can prevent the development of secondary complications.

## Acknowledgments

The authors would like to thank the staff of the Department of Pathology and Microbiology, ESIC Medical College and Hospital, Faridabad, India for their valuable cooperation.

## Authors’ contribution

Conception and design: S. R., M. P., J. T., M. J., A. D., T. CH., R. CH., and A. K. R. 

Acquisition of data: S. R., J. S., J. T., K. S., and Z. A. 

Drafting the article: K. S., S. R., J. T., and M. P. 

Final approval of the completed article: S. R., M. P., J. T., M. J., S. D., T. CH., R. CH., A. K. R., J. S., and Z. A.

All authors provided critical revisions for important intellectual content and also read and approved the final manuscript.

## Conflicts of interest

There is no conflict of interest.

## Financial disclosure

The authors declare no financial interest related to this study.
